# miRNA-21 is developmentally regulated in mouse brain and is co-expressed with SOX2 in glioma

**DOI:** 10.1186/1471-2407-12-378

**Published:** 2012-08-29

**Authors:** Jelena Põlajeva, Fredrik J Swartling, Yiwen Jiang, Umashankar Singh, Kristian Pietras, Lene Uhrbom, Bengt Westermark, Pernilla Roswall

**Affiliations:** 1 Department of Immunology, Genetics and Pathology, Rudbeck Laboratory, SE-751 85, Uppsala, SWEDEN; 2 Department of Medical Biochemistry and Biophysics, Karolinska Institutet, SE-171 77, Stockholm, SWEDEN

**Keywords:** miRNA, miR-21, Glioma, PDGF-BB, SOX2, Imatinib (Gleevec), RCAS/tv-a

## Abstract

**Background:**

MicroRNAs (miRNAs) and their role during tumor development have been studied in great detail during the last decade, albeit their expression pattern and regulation during normal development are however not so well established. Previous studies have shown that miRNAs are differentially expressed in solid human tumors. Platelet-derived growth factor (PDGF) signaling is known to be involved in normal development of the brain as well as in malignant primary brain tumors, gliomas, but the complete mechanism is still lacking. We decided to investigate the expression of the oncogenic miR-21 during normal mouse development and glioma, focusing on PDGF signaling as a potential regulator of miR-21.

**Methods:**

We generated mouse glioma using the RCAS/tv-a system for driving PDGF-BB expression in a cell-specific manner. Expression of miR-21 in mouse cell cultures and mouse brain were assessed using Northern blot analysis and *in situ* hybridization. Immunohistochemistry and Western blot analysis were used to investigate SOX2 expression. LNA-modified siRNA was used for irreversible depletion of miR-21. For inhibition of PDGF signaling Gleevec (imatinib mesylate), Rapamycin and U0126, as well as siRNA were used. Statistical significance was calculated using double-sided unpaired Student´s *t*-test.

**Results:**

We identified miR-21 to be highly expressed during embryonic and newborn brain development followed by a gradual decrease until undetectable at postnatal day 7 (P7), this pattern correlated with SOX2 expression. Furthermore, miR-21 and SOX2 showed up-regulation and overlapping expression pattern in RCAS/tv-a generated mouse brain tumor specimens. Upon irreversible depletion of miR-21 the expression of SOX2 was strongly diminished in both mouse primary glioma cultures and human glioma cell lines. Interestingly, in normal fibroblasts the expression of miR-21 was induced by PDGF-BB, and inhibition of PDGF signaling in mouse glioma primary cultures resulted in suppression of miR-21 suggesting that miR-21 is indeed regulated by PDGF signaling.

**Conclusions:**

Our data show that miR-21 and SOX2 are tightly regulated already during embryogenesis and define a distinct population with putative tumor cell of origin characteristics. Furthermore, we believe that miR-21 is a mediator of PDGF-driven brain tumors, which suggests miR-21 as a promising target for treatment of glioma.

## Background

Despite the discovery and extensive studies of a large number of microRNAs (miRNAs), the role and molecular mechanisms of their actions are still unclear [1]. miRNAs have been shown to be involved in tumor development [2,3]. The expression pattern and signature of certain miRNAs are important for the regulation of cell fate [4,5], and their expression signature has been shown to correlate with tumor initiation and progression indicating a prognostic and diagnostic potential [6,7]. We have focused on miR-21 that recently was identified as differentially expressed in a high number of solid tumors when comparing to normal tissue [8]. In addition miR-21 has been demonstrated to be up-regulated in a majority of human cell lines and tumor tissues such as glioblastoma [9], breast cancer [10], and chronic lymphocytic leukemia [11]. Based on knockdown studies, it has been proposed that miR-21 acts as an oncogene exerting its effect by down-regulating crucial apoptosis-related genes [9]. Furthermore, gain of function and loss of function of miR-21 in a transgenic mouse model for non-small-cell lung cancer showed that miR-21 behaved as a tumor promoter [12]. Likewise, in a transgenic mouse model for conditional expression of miR-21, a complete regression of B-cell lymphoma was observed upon withdrawal of miR-21 [13].

Glioblastoma multiforme (GBM) is the most frequent and most malignant (grade IV) form of adult glioma. GBMs are highly invasive and the median survival after diagnosis ranges from 9 months to 2 years [14,15]. Like in all cancers, glioma development is associated with uncontrolled proliferation and escape of regulatory control of the cell cycle [16]. Astrocytic gliomas of various grades have been shown to overexpress platelet-derived growth factor receptor alpha (PDGFR-α), whereas both PDGF-AA and PDGF-BB have been consistently found in high grade gliomas (grade III and IV) only, generating autocrine stimulation [17]. Despite the extensive increase in knowledge in the past decade, the clinical outcome of human gliomas has remained constant, rationalizing the need for further studies.

In general, there is a lack of knowledge concerning the expression and function of miRNAs during normal development. We decided to investigate the expression of miR-21 during normal brain development in mice. Interestingly, miR-21 was indeed shown to be expressed already at embryonic day E18, displaying a sustained expression also in the newborn brain. This expression was in some defined areas overlapping with SOX2 expressing cells. miR-21 and to a large extent also SOX2 expression were lost in the adult brain, indicating a co-regulation. However, mouse gliomas show high expression of miR-21. Furthermore, inhibition of PDGF signaling using Imatinib (Gleevec), Rapamycin and U0126, significantly reduced miR-21 levels in mouse glioma primary cultures. Upon siRNA-mediated knockdown of miR-21, the levels of SOX2 in both mouse glioma cell lines and human glioblastoma cell lines strongly decreased. This was further strengthened by *in situ* hybridization on mouse brains revealing that the expression pattern of miR-21 was specific to tumor areas and strongly overlapped with areas staining positive for SOX2. Our data propose that the embryonic expression pattern of miR-21 is maintained or re-established during initiation and progression of glioblastoma.

## Methods

### Ethics Statements

All animal experiments were approved (ethical approval number C18/6, 2006-02-24) and performed in accordance with the rules and regulations of the Ethical Committee for Animal Experiments in Uppsala (Sweden). Patient samples were obtained following approval by the Regional Ethical Review Board in Uppsala (ethical approval number 2007/353, 2008-03-12). Patients provided written informed consent for the collection of samples.

### Cell culture and tumor samples

Human glioma cell lines U343MG, Cl2:6, U87MG, U1242MG, U251MG, U373MG, U2987MG were previously established in our laboratory [18-20]. Cells were cultured in Minimum Essential Medium (MEM) supplemented with 10% fetal bovine serum (FBS), 2 mM L-glutamine and 100 units/ml penicillin and 0.1mg/ml streptomycin (Sigma Aldrich, St Louis, MO).

Mouse glioma cell cultures were established from RCAS/PDGFB-induced gliomas in a wild type, p16^Ink4a−/−^, p19^Arf−/−^ or p16^Ink4a−/−^/p19^Arf−/−^ background [21-27]. The t-va retroviral receptor is expressed in transgenic mice under the control of the nestin or the GFAP promoter, addressing neural/glial progenitor cells and astrocytes, respectively. In brief, an immortalized chicken fibroblast cell line, DF1 (American Type Culture Collection), producing RCAS/PDGF-B virus particles, was injected intracerebrally in newborn mice [28,29]. At sign of brain tumor, mice were euthanized and the brains were aseptically dissected out. The brains were cut with a coronal section at the injection site, and the anterior part was mechanically disrupted and used for establishing cell cultures whereas the posterior part was formalin-fixed and used for paraffin sectioning. The mouse glioma cells, the human fibroblast cell line 1064SK and LN18 glioma cells [30], kindly provided Dr Nicolas de Tribolet, Lausanne University, were cultured in Dulbeco’s Modified Essential Medium (DMEM) supplemented with 10% fetal bovine serum (FBS), 4mM L-glutamine and 100 units/ml penicillin and 0.1 mg/ml streptomycin (Sigma Aldrich). All cells were grown at 37°C with 5% CO_2_. Embryos were collected, formalin-fixed and paraffin embedded.

### Human and mouse glioma-derived cancer-initiating cell (GICs) cultures

Low passage human glioma cell culture U3001MG was recently established in our group [31]. Fresh tumor samples were obtained from adult patients during operative procedure. The tumor was graded at the Uppsala University Hospital by a neuropathologist according to World Health Organization (WHO) guidelines. After the primary sphere formation, the spheres were seeded onto dishes coated with ECM gel (Sigma Aldrich) and cultured as adherent cells as described before [32] in complete BTIC media, containing DMEM/F12 Glutamax (GIBCO/Invitrogen, Carlsbad, CA), 10mM HEPES (Sigma Aldrich), 25 μg/ml insulin, 100 μg/ml transferrin, 20 nM progesterone, 10 μM putrescine, 30 nM selenite, 1% B27 (Invitrogen), 100 units/ml penicillin and 0.1 mg/ml streptomycin (Sigma Aldrich), 20 ng/ml EGF and FGF2 (Peprotech Rocky, Hill, NJ, USA).TC1, low passage mouse glioma-derived cancer-initiating cells (GICs) were recently established in our lab, these were cultured in complete BTIC media, excluding EGF and FGF, as neurospheres [33].

### RNA preparation

After starvation in serum-free media, 1064SK cells were treated with PDGF-BB (10 mg/ml). Unless otherwise stated, total RNA was extracted from human and mouse cell lines using TRIzol reagent (Invitrogen) according to the manufacturer’s instructions. In short, cells were washed with PBS, scraped off and spun down. The pellet was subjected to TRIzol reagent and homogenized before chloroform extraction. RNA was precipitated with isopropanol and washed in 70% EtOH, before being eluted in DEPC-H_2_O.

After starvation in serum-free media, Nestin p19^Arf−/−^ cells were treated with the following inhibitors for 24 hours (h): Imatinib mesylate (20 μM, BioVision, Mountain View, CA), UO126 (10 μM, Cell Signaling Technology, Danvers, MA), LY294002 (10 μM, Cell Signaling Technology), Rapamycin (100 nM, Cell Signaling Technology). Following inhibition of PDGF signaling, small RNA fraction was extracted using MirVana Isolation Kit (Applied Biosystems, Carlsbad, CA) according to manufacturer’s instructions. Briefly, cell lysate was once extracted with Acid-Phenol:Chloroform and further enriched for the small RNA fraction over a glass-fiber filter. Finally, the RNA was eluted in DEPC-H_2_O containing 1% elution solution provided with the kit.

### *In situ* hybridization

After deparaffinization of coronal sections of mouse brain, the tissues were subjected to pepsin (1.3 mg/ml, Sigma Aldrich) for 30 min. After washing in PBS the slides were submerged in 99.7% ethanol and air-dried. Hybridization was performed in a humidified chamber at 37°C for 16-18 h, with a digoxigenin-labelled locked nucleic acid (LNA) modified oligonucleotide (Integrated DNA Technologies, Leuven, Belgium) diluted in Enzo *In situ*-hybridization buffer (Enzo Life Sciences, Inc., Farmingdale, NY) to the concentration of 2 pmole/μl. After hybridization, slides were rinsed at 4°C in washing buffer (Enzo Life Sciences, Inc.) and subjected to anti-digoxigenin alkaline phosphatase Fab (Roche, Basel, Switzerland) at 37°C for 30 min. After incubation in AP detection reagent (Enzo Life Sciences, Inc.) the slides were incubated with NBT/BCIP reaction mixture (Enzo Life Sciences, Inc.) in a humidified chamber at 37°C. The slides were counterstained with Red counter stain (Enzo Life Sciences, Inc.) and then washed in PBS, 100% Ethanol and Xylene. The slides were mounted in Pertex (Leica Microsystems, Kista, Sweden).

### Transfections

LNA modified antisense miR-21 (5´-TCAACATCAGTCTGATAAGCTA-3´) and antisense enhanced green fluorescence protein (EGFP) (5´-AAGGCAAGCUGACCCUGAAGU-3´) used as a negative control, were purchased from Integrated DNA Technologies. Cells were subconfluently seeded in petri-dishes in antibiotic-free culture media. Twenty-four hours after seeding the cells were transfected with 50 nM LNA-miR-21 and si-EGFP with Lipofectamine RNAiMAX (Invitrogen, San Diego, CA) in serum-free medium. After 6 h the culture medium was changed to regular medium containing antibiotics and serum. Forty-eight hours after transfection, the cells were collected for RNA extraction. Cells were transfected with control siRNA or siRNA against human PDGF-BB (Dharmacon, Lafayette CO) at a concentration of 50 nM using Dharmacon 2 (Dharmacon). Seventy-two h post transfection, cells were collected. Transfection efficiency was determined to be significant using quantitative real-time PCR as previously described [34]. Human *PDGFB* expression was normalized to mouse *Hprt* (data not shown).

### Northern blot analysis

Samples of total RNA (5-10 μg/lane) were electrophoresed on a 15% TBE-Urea gel (NuPAGE, Invitrogen, San Diego, CA) under denaturing conditions according to the protocol supplied by the distributor. The RNA was transferred to a Hybond N^+^ membrane (GE Healthcare, Uppsala, Sweden). Hybridization was performed using 10 mM Na_2_HPO_4_, 10 mM NaH_2_PO_4_, 0.75 M NaCl, 75 mM Sodium Citrate, 0.02% Albumin, 7% SDS, 0.02% Ficoll 400 solution. *miR21* or *U6 snRNA* DNA oligos were labeled in the 5´ end with [γ-^32^P]ATP using T4 Polynucleotide Kinase (New England Biolabs, Ipswich, MA), purified with G-25 MicroSpin Columns (GE Healthcare) and used in the hybridization step (42°C, 16-18 h). After being washed in 2xSSC 0.1% SDS the membrane was exposed to an X-ray film (Hyperfilm ECL, Amersham Biosciences).

### Quantitative real-time PCR

Stem-loop reverse transcription for miR-21 was performed using TaqMan® MicroRNA Reverse Transcription Kit according to manufacturers’ description (Applied Biosystems, Carlsbad, CA). In short, RNA was reverse transcribed into cDNA. After dilution quantitative RT-PCR was performed using Stratagene Mx 3001P (Stratagene, La Jolla, CA) and TaqMan®MicroRNA Assays for miR-21 together with the TaqMan® Universal PCR Master Mix (Applied Biosystems). All samples were run in triplicates for 45 cycles in a two-step PCR at 95°C and 60°C. To calculate relative gene expression, the comparative threshold cycle (C_T_) method 2^-ΔΔCT^ was applied where C_T_ is defined as the fractional cycle number at which the fluorescence passes the fixed threshold [35]. Values of miR-21 were normalized to expression of miR-16 (set to 1), and the relative expression was quantified. U6 was also used as a control gene, showing similar results as miR-16.

### Immunoblot analysis

Cells were subjected to a lysis buffer (1% Triton X-100, 150 mM NaCl, 10 mM Tris–HCl pH 7.4, 1 mM EGTA, 1 mM EDTA, 0.5% NP-40, 35 ng/ml phenylmethylsulphonyl fluoride, 1.4 μg/ml aprotinin, 1 mM Na_3_VO_4_, 1 mM ZnCl_2_, 50 mM Na_2_MoO_4_). Protein concentration was determined using BSA Protein Assay according to the manufacturer´s instructions (Pierce Chemical Co, Rockford, IL). Protein samples (5-10 μg per lane) were subjected to a sodium dodecyl sulphate-polyacrylamide gel electrophoresis (SDS-PAGE; NuPAGE 4-12% Bis-Tris Gel, Invitrogen) according to the protocol supplied by the distributor. Proteins were transferred to a nitrocellulose filter (Hybond ECL, Amersham Biosciences, Uppsala, Sweden). The filters were blocked and subjected to antibodies in 5% dry milk in TBS-T (10 nm Tris-base, 0.15 M NaCl, pH 7.7 and 0.1% Tween). Filters were developed using substrate solution (Chemoluminiscence, Super Signal, Pierce Chemical Co.) on x-ray films (Hyperfilm ECL, GE Healthcare). The antibodies used were: Cleaved Caspase-3 (Asp175) (Cell Signaling Technology), GAPDH (Cell Signaling Technology, Danvers, MA), SOX2 (Abcam, USA). Before being used again the filters were stripped in a solution containing 100 mM β-mercaptoethanol, 2% SDS and 62.5 mM Tris–HCl at pH 6.7.

### Immunohistochemical analysis

Paraffin embedded mouse brains were sectioned in 5 μm and adhered to glass slides, deparaffinized and pressure boiled in citric buffer. Ultra Vision LP detection system (Thermo Fisher Scientific, Fremont, CA) was used according to manufacturer’s instructions. Briefly, slides were incubated with Hydrogen Peroxidise Block, followed by Ultra V Block treatment. Antibodies were diluted in 5% normal goat serum and incubated over night (rabbit anti-mouse SOX2 and rabbit anti-mouse OLIG2 (Millipore, Billerica, MA). Primary antibody enhancer, HRP polymer and DAB Plus Chromogen were used to visualize the staining. Slides were counterstained with hematoxylin and mounted using Immu-mount (Thermo Fisher Scientific, Fremont, CA). Images were taken using a Zeiss Observer Z1 microscope and an AxioCam HRc Zeiss camera.

### AnnexinV analysis

Cells cultured in duplicates in two individual experiments were treated with LNA-miR-21 or si-EGFP (previously described) for 48 h counting from addition of the si-RNA. All cells, attached and detached were collected and washed in PBS. To investigate apoptosis annexinV Alexa Fluor®647 in combination with PI were added (Vybrant® Apoptosis Assay Kit, Molecular Probes^TM^ Invitrogen detection technologies). Apoptosis was analyzed by flow cytometry (BD FACS^TM^ LSRII).

### Statistical analysis

Statistical significance was calculated using double-sided unpaired Student´s *t*-test. Significance was calculated from two independent experiments in the PDGFR signaling inhibitory trial and five independent experiments in the si-PDGF-BB trial. The measurements are presented as mean fold changes ± standard error of the mean (SEM).

## Results

### miR-21 is expressed during brain development but is absent in adult brain

**Figure 1:**
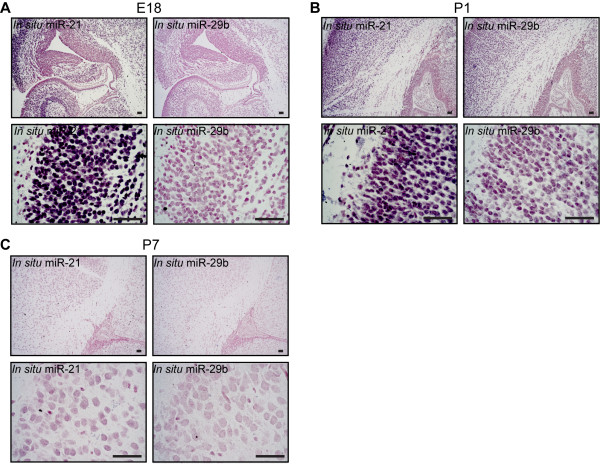
**miR-21 is expressed during embryonic development.***In situ* hybridization with a digoxigenin-labeled probe, showing differential expression of miR-21 in normal E18 (**A**), newborn (P1) (**B**) and P7 (**C**) *Gtv a wild type* mouse brain. miR-21 could be seen in hippocampus as well as areas close to the ventricle. A digoxigenin miR-29b was used as an independent miRNA control. Scale bar is indicated in the figure and represents 50 μm.

To investigate the involvement of miR-21 during embryogenesis we analyzed its expression pattern in the developing mouse brain, using *Gtv-a* wild type mice. Coronal tissue sections of paraffin embedded normal brain from embryonic day 18 (E18), were subjected to *in situ* hybridization, which revealed that miR-21 was highly expressed in the hippocampus and the outer rim of the cortex (containing martinotti, pyramidal, and stellate cells) as shown in Figure 1A. High expression was also found in the same areas in newborn mouse brain (Figure 1B). However, at postnatal day 7 (P7) and onwards miR-21 expression was strongly reduced and no expression was found in the adult brain (Figure 1C, and data not shown). miR-29b was used as an independent control miRNA. Thus, miR-21 expression appears to be developmentally regulated and absent in adult brain tissue.

### Expression of SOX2 overlaps with miR-21 expression during embryogenesis

**Figure 2:**
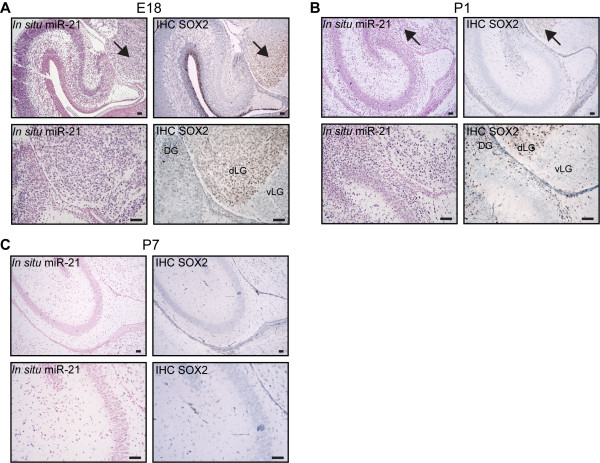
**Overlapping expression of SOX2 and miR-21.** IHC and *in situ* hybridization showing the expression pattern of SOX2 and miR-21, respectively. miR-21 expression in Gtv a wild type mouse brain at different developmental stages E18 is shown in **A**), P1 in **B**) and P7 in **C**). Scale bar is indicated in the figure and represents 50 μm. Arrow indicates overlapping expression of SOX2 and miR-21. Abbreviations: dorsal lateral geniculate nucleus (dLG), ventral lateral geniculate nucleus (vLG), dentate gyrus (DG).

Knowing that SOX2 is required to maintain cellular pluripotency in the developing embryo [36], we decided to investigate the expression pattern of miR-21 during mouse brain development in relation to SOX2 expression, using *Gtv-a* wild type mice. We used immunohistochemical (IHC) staining and *in situ* hybridization to demonstrate that SOX2 and miR-21 showed overlapping expression at E18 and P1 (Figure 2A, 2B). Dorsal lateral geniculate nucleus (dLG) and dentate gyrus (DG) represent areas with a large percent of double positive cells. However, a heterogeneity could be seen with a clear boundary distinguishing cells in the ventral lateral geniculate nucleus (vLG) that are negative for SOX2, as previously described [37], but positive for mir-21. The expression of SOX2 as well as miR-21 was substantially decreased at P7 (Figure 2C), also indicating a co-regulation.

### miR-21 is highly expressed in mouse glioma cells and tissue

**Figure 3:**
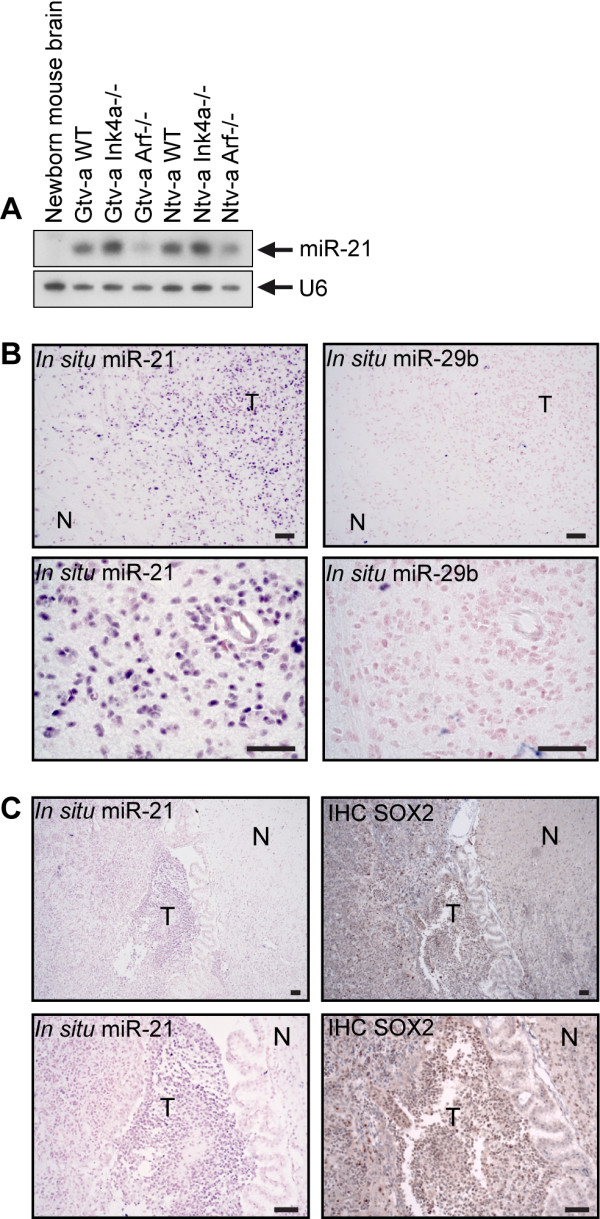
**Increased levels of miR-21 in mouse glioma cell lines and glioma. A**) Compared to normal mouse brain the different mouse primary glioma cultures had an increased expression of miR-21 as measured by Northern blot. U6 was used as a loading control. In **B**) *in situ* hybridization of miR-21 in the tumor area of a *Gtv-a* p16^Ink4a−/−^/p19^Arf−/−^ mouse brain is presented, using a digoxigenin-labeled miR-29b probe as an independent miRNA control. In **C)** a *Gtv-a* p16^Ink4a−/−^/p19^Arf−/−^ mouse brain could be seen, showing miR-21 expression specifically to the tumor area adjacent to the ventricle and the choroid plexus. T = tumor, and N = normal. Scale bar is indicated in the figure and represents 50 μm.

Previous studies describing miR-21 as an oncogene [3] prompted us to generate experimental gliomas using the RCAS/tv-a mouse model system [28]. Through intracerebral injection of cells producing RCAS/PDGF-B virus, gliomas were induced in a cell specific manner in newborn *Ntv-a* and *Gtv-a* mice with either wild type, p16^Ink4a−/−^, p19^Arf−/−^ or p16^Ink4a−/−^/p19^Arf−/−^ background. The PDGFB-induced gliomas are generated by an autocrine/paracrine stimulation and expansion of PDGFR-α positive glial progenitor/neural stem cells present in the newborn mouse brain [38,39]. We hypothesize that the aberrant expression of miR-21 in glioma cells may exist in the normal progenitor and become re-activated when that cell is targeted for tumor development, knowing that some features of embryogenesis are recapitulated in the tumor setting. As in previous studies [40] tumors mimicking human gliomas appeared after four to twelve weeks, depending on the genetic background of the mouse. Primary mouse glioma cell cultures were established and Northern blot analysis showed that expression levels of miR-21 varied between these cultures, although they all showed an increased level as compared to normal mouse brain (Figure 3A). The p16^Ink4a−/−^ cells had the highest expression, p19^Arf−/−^ the lowest, whereas an intermediate expression was found in wild type cells. Double knockout for both tumor suppressors, p16^Ink4a^ and p19^Arf^, had similar expression as p16^Ink4a^ single knockout (data not shown). To further investigate the expression pattern of miR-21 *in vivo*, coronal tissue sections from paraffin embedded tumor-bearing *Gtv-a* p16^Ink4a−/−^/p19^Arf−/−^ mouse brains were subjected to *in situ* hybridization. miR-21 positive cells were restricted to the tumor areas, showing no expression in the adjacent normal cells (Figure 3B and 3C).

### Overlapping expression pattern of miR-21 and SOX2 in mouse glioma

**Figure 4:**
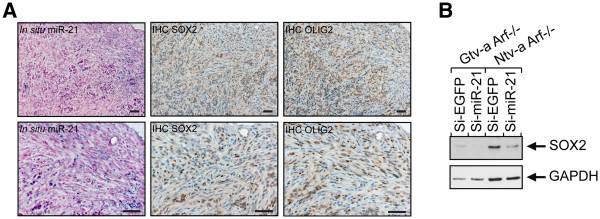
***In situ *hybridization and IHC, showing similar expression pattern for miR-21, SOX2, and the tumor cell marker OLIG2.** Inhibition of miR-21 causes a decrease in SOX2 expression. **A**) Serial paraffin coronal sections of a *Gtv-a* p16^Ink4a−/−^/p19^Arf−/−^ mouse brain showing overlapping expression of miR-21, SOX2, and OLIG2. Scale bar is indicated in the figure and represents 50 μm. Western blot analysis, demonstrating a decreased expression of the stem cell transcription factor SOX2, after LNA-miR-21 in p19^Arf−/−^ mouse primary glioma cultures is shown in **B**). GAPDH was used as a loading control.

Encouraged by our previous finding of the overlapping expression pattern of miR-21 and SOX2 in the normal developing mouse brain, we performed IHC staining of mouse glioma. It showed that the previously observed overlap of miR-21 and SOX2 expression was even more pronounced, exhibiting a total overlap in the tumor area (Figure 3C). On serial sections from one individual mouse brain we could see that SOX2 was also co-expressed with OLIG2 (Figure 4A), which is a surrogate marker for brain tumor cells [41,42].

### miR-21 knockdown is followed by a decrease in SOX2 expression

To investigate the interplay between miR-21 and SOX2 further we performed a target screen based on bioinformatics ( http://www.TargetScan.org) which suggested SOX2 to be a potential target of miR-21 as opposed to our *in vivo* finding of an overlapping expression pattern. We next analyzed the effect of miR-21 on SOX2 in p19^Arf−/−^ mouse glioma primary cultures after repression of miR-21 using locked nucleic acid (LNA)-modified antisense miR-21. Suppression of miR-21 resulted in a decrease of SOX2 in glioma cell cultures derived from both Gtv-a and Ntv-a mice (Figure 4B). We therefore conclude that miR-21, directly or through an intermediate target, participates in the up-regulation of SOX2 in these cells.

### PDGF-BB induces miR-21 expression

**Figure 5:**
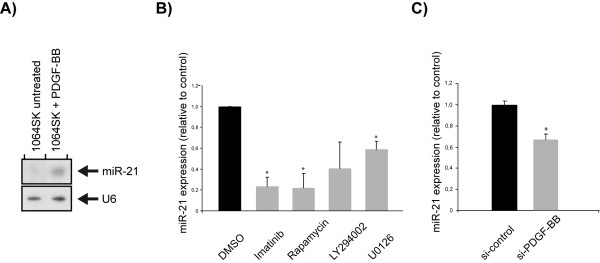
**PDGF-BB stimulation of normal fibroblasts resulted in increased expression of miR-21 whereas inhibition of PDGF signaling in glioma cells caused a decrease of miR-21.** Inhibition of PDGF signaling causes a decrease of miR-21 in a glioma cell culture and glioma initiating cells cultured as spheres, respectively. (**A**) Up-regulation of miR-21 expression could be seen, using Northern blot, in 1064SK cells after treatment with PDGF-BB. U6 was used as a loading control. (**B**) miR-21 levels, normalized to miR-16, after inhibition of PDGF signaling in p19^Arf−/−^ cells with a panel of inhibitors. Bars represent mean values from two independent experiments. SEMs are indicated in the figure and * indicates statistical significance (p < 0.05) from Student´s *t*-test. (**C**) The expression level of miR-21 after si-PDGF-BB was analyzed by qPCR in TC1 cells. miR-21 values were normalized to miR-16 and represent mean values from five independent experiments. SEMs are indicated in the figure and * indicates statistical significance (p < 0.05) from Student´s *t*-test.

In order to investigate the mechanism behind miR-21 expression we used a human fibroblast cell line, 1064SK. 1064SK cells with low basal levels of miR-21 were subjected to PDGF-BB stimulation resulting in a strong increase in miR-21 expression (Figure 5A). miR-21 expression is thus indirectly or directly induced by PDGF signaling.

### Inhibition of PDGF signaling causes down-regulation of miR-21

If the sustained and increased expression of miR-21 in the experimental glioma cells is caused by an up-regulation of PDGF signaling, we would expect to find a down-regulation of miR-21 upon inhibition of PDGF signaling. By using inhibitors for the PDGFR itself and for known targets downstream of the PDGF receptor, we could verify that miR-21 was indeed decreased (Figure 5B). Treatment of p19^Arf−/−^ mouse glioma primary cultures with Gleevec (imatinib mesylate), Rapamycin and U0126, all significantly reduced the miR-21 levels, as shown by qPCR. A reduction could also be shown after LY294002 (inhibiting PI3 kinase) treatment, but this was not significant. The efficiency of the inhibitors in decreasing their target´s protein phosphorylation was observed compared to controls using Western blotting (data not shown). We also investigated the effect on miR-21 expression in glioma-derived cancer initiating cells (GICs) cultured as spheres derived from PDGFB-induced tumors in neonatal Gtv-a mice [33]. When PDGF-BB expression was inhibited by siRNA against *PDGF-B* we found a significant decrease in miR-21 expression as shown by qPCR (Figure 5C). The reduction in miR-21 expression could be coupled to a decrease in SOX2 expression, loss of tumor-initiating ability of the cells and induction of oligodendrocyte differentiation [33].

### Elevated expression of miR-21 in human glioblastoma cell lines

**Figure 6:**
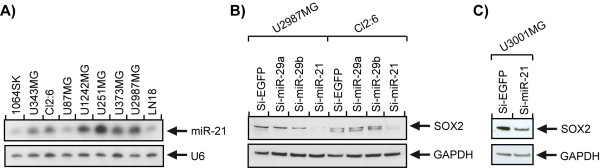
**Elevated expression of miR-21 in human glioblastoma cell lines and down regulation of SOX2 upon miR-21 knock down. A**) Human glioblastoma cell lines revealed a higher expression of miR-21 compared to the normal fibroblast cell line, 1064SK, as shown by Northern blot analysis. U6 was used as a loading control. Western blot analysis showing a decreased expression of SOX2, after LNA-miR-21 in the human glioblastoma cell lines U2987MG and Cl2:6 (**B**), and in human glioblastoma primary culture, U3001MG (**C)**. GAPDH was used as a loading control.

To verify our findings on mouse glioma, we used a panel of human glioblastoma cell lines and analyzed them for miR-21 expression by Northern blot analysis. All cell lines expressed miR-21 at considerably higher levels than 1064SK used as control, in accordance with previous publications [9]. The extent of miR-21 expression varied between different cell lines, U251MG revealed the highest expression and U87MG and LN18 the lowest (Figure 6A). Two of the cell lines, U2987MG and Cl2:6, showed *in vitro* expression of SOX2 [34]. Western blot analysis of these cell lines demonstrated a decrease in SOX2 expression upon addition of LNA-miR-21 (Figure 6B), which confirms the data obtained from the mouse glioma cell lines. A similar effect was seen in a low passage human glioma cell culture, U3001MG, grown in serum-free stem cell medium (Figure 6C).

### Growth inhibition and apoptosis were induced by inhibition of miR-21

**Figure 7:**
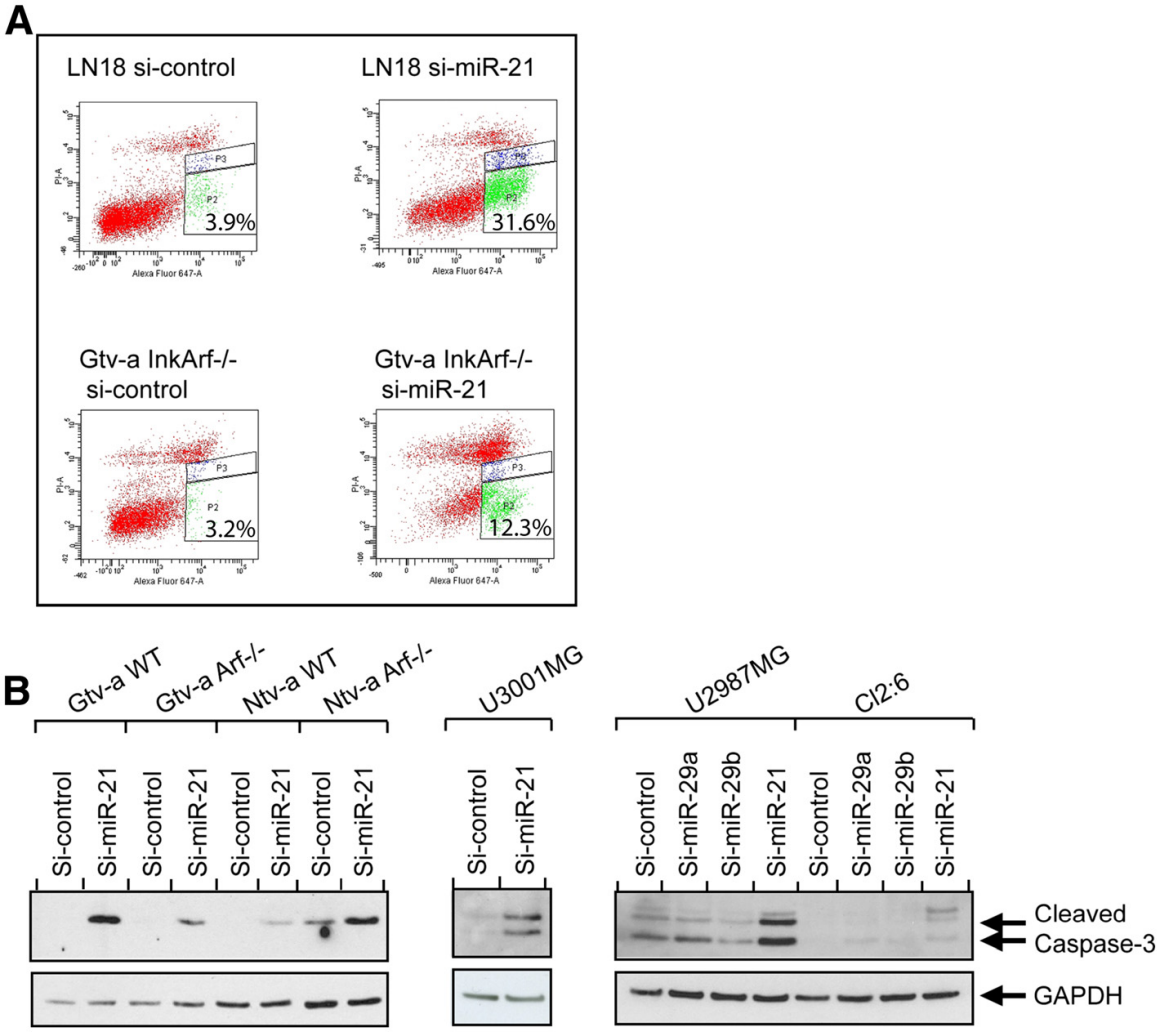
**Inhibition of miR-21 mediated apoptosis in both mouse and human tumor cells. A**) AnnexinV staining showing that both mouse and human glioma cells undergo apoptosis upon miR-21 inhibition by using LNA-miR-21 as compared to si-control. **B**) A panel of mouse glioma cell lines (left), the human glioblastoma primary culture U3001MG (center), and the human glioblastoma cell lines Cl2:6 and U2987MG (right) showed an increased level of cleaved Caspase-3 after LNA-miR-21 as compared to si-control and two independent miRNAs (miR-29a and miR-29b) as shown by Western blot analysis. GAPDH was used as a loading control.

Suppression of miR-21 in human cell lines has previously been shown to result in apoptosis [9]. We investigated whether a similar response was elicited in primary mouse tumor cultures. Several apoptotic measurements were performed upon miR-21 knockdown. Cell cycle analysis revealed a strong increase in the SubG1-fraction (data not shown). Furthermore, AnnexinV-staining showed a five-fold increase in the number of apoptotic cells after repression of miR-21 by LNA-miR-21 both in an p16^Ink4a^/p19^Arf^ double knockout mouse glioma cell culture as well as in the human glioblastoma cell line LN18 (Figure 7A). The induction of apoptosis could also be demonstrated by measuring the amount of cleaved Caspase-3, an apoptotic marker. Western blot analysis supported induction of apoptosis by showing that loss of miR-21 through LNA-miR-21 treatment increased the amount of cleaved and activated Caspase-3. In Figure 7B the result for a panel of primary mouse glioma cultures, the low passage human glioma cell culture (U3001MG), and two human glioblastoma cell lines (Cl2:6 and U2987MG) are presented.

## Discussion

Our data show that miR-21 is expressed during normal embryogenesis and is tightly regulated in normal mouse development. In mouse brain at E18, miR-21 was highly expressed in areas known to contain a large number of neural/glial progenitor cells, viz. hippocampus, dentate gyrus and outer rim of the cortex. This pattern of miR-21 expression was sustained in the newborn brain but at P7, the expression was abolished and no expression could be found in the adult brain. The finding that miR-21 is expressed in the immature brain, but not in the adult tissue, indicates that miR-21 is of developmental importance with a controlled and restricted expression. Its overlapping expression with SOX2 is further suggestive since SOX2 has been demonstrated to be involved in the proliferation and/or maintenance of neural stem cells in the developing brain [43,44], indicating miR-21 to share these functions. We went on investigating brain tumors and showed that miR-21 is overexpressed in glioma tissue and primary cultures established from RCAS/PDGFB-induced mouse gliomas, mimicking human high grade gliomas. The expression of miR-21 in PDGFB-induced mouse glioma was confined to the tumor areas as shown by *in situ* hybridization. SOX2 has previously been shown to be involved in the maintenance of stem cell properties and prevention of differentiation [45]. When performing IHC staining of mouse brain tumors, an almost complete overlap between SOX2 and miR-21 expression could be seen. And although the role of miRNAs in stem cell biology has not been fully explored, there is emerging evidence suggesting posttranscriptional regulation of genes as an important step in stem cell biology [46,47]. Tumor-initiating cells or cancer stem cells have been found in many types of cancers. These cells have been thought to represent the radiotherapy and drug-resistant cell population [48,49]. When studying embryonic stem cells, siRNA against the DNA-binding protein REST resulted in an increased expression of miR-21 accompanied by a reduced expression of SOX2 and thereby a suppression of self-renewal [50,51]. In this paper we reveal that siRNA-mediated knockdown of miR-21 led to a significant reduction of SOX2 in both mouse and human glioma cells. The discrepancy between Singh *et al.*[51] and our results indicate that miRNA expression pattern, as well as downstream effects, differ in different cell types depending on cellular context and the available mRNA targets. Our findings suggest that miR-21 indirectly sustains the SOX2 expression and thereby is involved in the maintenance of the glial progenitor/stem cell phenotype. These functions are then recapitulated in the glioma cells, in the present experimental mouse glioma system, most likely caused by induced PDGF-BB expression. One might consider that PDGFB-induced tumor development involves activation of single or multiple signaling pathways leading to continuous expression of miR-21, followed by an increased expression of SOX2, thereby keeping the cell in a progenitor/stem cell stage which protects them from undergoing apoptosis. Recent data from Bao *et al.* support such a view of miR-21 as a mediator of the cancer stem cell phenotype [52]. Upon direct knockdown of miR-21 in tumor cells, addicted to miR-21 expression, apoptosis is induced [9] and Figure 7 in this paper. However, one cannot exclude other mechanisms for tumor inhibition upon miR-21 suppression. For example, PDGF-B inhibition that causes down-regulation of miR-21 (Figure 5C) is also known to differentiate PDGFB-driven mouse brain tumor cells along the oligodendrocyte lineage [33]. Specific mechanisms for miR-21 regulation have been suggested in breast cancer as well as lung cancer [53,54]. Here, we investigated the role for the PDGF signaling pathway on miR-21 regulation in glioma. By using a panel of inhibitors of PDGF signaling, we could conclude that PDGF-BB and PDGFR-α signaling drives miR-21 expression in primary mouse glioma cultures. Likewise, si-PDGF-BB treatment of a primary mouse glioma sphere culture resulted in a down-regulation of miR-21, followed by a decrease in the number of spheres, further strengthening the notion that miR-21 is indeed driven by PDGF-BB signaling in these tumor cells. Shao *et al.* recently described PDGF-AA and PDGF-BB to regulate a number of miRNAs in the glioblastoma cell line U118, highlighting the role of PDGF signaling in the alteration of miRNAs and tumor development and progression [55].

Specific mechanisms for miR-21 regulation have been suggested in breast cancer as well as lung cancer [53,54]. Here, we investigated the role for the PDGF-signaling pathway on miR-21 regulation in glioma. By using a panel of inhibitors of PDGF-signaling, we could conclude that PDGF-BB and PDGFR-α signaling drives miR-21 expression in primary mouse glioma cultures. Likewise, si-PDGF-BB treatment of a primary mouse glioma sphere culture resulted in a down-regulation of miR-21, followed by a decrease in the number of spheres, further strengthening the notion that miR-21 is indeed driven by PDGF-BB signaling in these tumor cells. Shao *et al.* recently described PDGF-AA and PDGF-BB to regulate a number of miRNAs in the glioblastoma cell line U118, high-lighting the role of PDGF-signaling in the alteration of miRNAs and tumor development and progession [55].

## Conclusions

We show that miR-21 and SOX2 are co-expressed during mouse brain development and subsequently down-regulated in the adult mouse brain. An elevated expression of miR-21 was found in PDGFB-induced mouse glioma. Knockdown of miR-21 with an LNA-modified siRNA or suppression of miR-21 through PDGF inhibition was accompanied by a decrease in SOX2. Our data suggest that miR-21 regulates SOX2 and is important in maintaining PDGF-driven brain tumors that constitute the large proneural subgroup of human malignant glioma [56]. The recently published data revealing that withdrawal of miR-21 in a mouse model leads to complete regression of tumors [13], make miR-21 a promising therapeutic target, particularly in glioblastoma where effective treatment modalities are still lacking.

## Competing interests

The authors declare that they have no competing interest.

## Authors’ contribution

JP carried out the qPCR studies, siRNA experiments, WB, IHC. YJ performed si-PDGF-BB experiments on GICs. US performed siRNA experiments. PR performed the *in vivo* studies and generation of glioma cell cultures, *in situ* hybridization, siRNA experiments, NB, WB, IHC and apoptotic assays. JP, FJS, US, KP, LU, BW, PR participated in the design of the study and coordination and helped to draft the manuscript. BW and PR wrote the manuscript. All authors read and approved the final manuscript.

## Pre-publication history

The pre-publication history for this paper can be accessed here:

http://www.biomedcentral.com/1471-2407/12/378/prepub
